# Event as the central construal of psychological time in humans

**DOI:** 10.3389/fpsyg.2024.1402903

**Published:** 2024-09-18

**Authors:** Sandra Stojić, Zoltan Nadasdy

**Affiliations:** ^1^Doctoral School of Psychology, ELTE Eötvös Loránd University, Budapest, Hungary; ^2^Institute of Psychology, ELTE Eötvös Loránd University, Budapest, Hungary; ^3^Department of Psychology, University of Texas at Austin, Austin, TX, United States; ^4^Zeto, Inc., Santa Clara, CA, United States

**Keywords:** events, time perception, event cognition, duration, ordinality, cognitive development

## Abstract

Time is a fundamental dimension of our perception and mental construction of reality. It enables resolving changes in our environment without a direct sensory representation of elapsed time. Therefore, the concept of time is inferential by nature, but the units of subjective time that provide meaningful segmentation of the influx of sensory input remain to be determined. In this review, we posit that events are the construal instances of time perception as they provide a reproducible and consistent segmentation of the content. In that light, we discuss the implications of this proposal by looking at “events” and their role in subjective time experience from cultural anthropological and ontogenetic perspectives, as well as their relevance for episodic memory. Furthermore, we discuss the significance of “events” for the two critical aspects of subjective time—duration and order. Because segmentation involves parsing event streams according to causal sequences, we also consider the role of causality in developing the concept of directionality of mental timelines. We offer a fresh perspective on representing past and future events before age 5 by an egocentric bi-directional timeline model before acquiring the allocentric concept of absolute time. Finally, we illustrate how the relationship between events and durations can resolve contradictory experimental results. Although “time” warrants a comprehensive interdisciplinary approach, we focus this review on “time perception”, the experience of time, without attempting to provide an all encompassing overview of the rich philosophical, physical, psychological, cognitive, linguistic, and neurophysiological context.

## 1 Meaning of events (in time)

In their seminal paper about event structure, Zacks and Tversky (2001) describe events as segments of time at a given location that, from an observer's perspective, have a defined beginning and an end. This description assumes the concept of uni-directionality and observable time with well-defined beginnings and end points encompassing segments. However, how the concept of time is constructed, especially before events are defined, remains elusive. This is the central question we address in this review. We argue that events are defined together with a primordial concept of time that enable segmentation of the stream of sensory input into percepts that form episodes, the building blocks of episodic memory.

Throughout history, events have been conceptualized within different frameworks but are most frequently discussed in analogy to objects (e.g., Zacks and Tversky, 2001; Casati and Varzi, 2008; De Freitas et al., 2014; Yousif and Scholl, 2019, as cited in Yates et al., 2023) in a way that “events serve to discretize time in the same manner the objects discretize the space” (Yates et al., 2023, p. 1) (for an overview of other event frameworks, under which the events are interpreted “as the consequences of prediction error” or “inferred causal structure,” see Yates et al., 2023, and for a brief overview of several theoretical models considering events as focal features of temporal cognition, see Table 1).

**Table 1:** While the passage of time is not directly detected by any sensory system, change is a constant and ever-present source of temporal information. The idea that the number of events in memory is related to how we perceive the passage of time was first put forward by James (1890). Here, we provide a brief overview of only several out of many prominent theoretical models that relied on the events, i.e., change, as the central construal of time, and we provide their explication concerning the perception of duration. In brief, in all these models, the more changes that occur during a given period of time, the longer the retrospective estimation of that segment is.

**References**	**The role of the events in the context of the novel findings**
Sturt (1925)	“*In estimating time, we rely on the amount of mental content experienced during that time... Time which has been filled by many thoughts appears longer whereas time occupied by few thoughts appears shorter. “*
Fraisse (1964)	“*…the duration judgments are primarily based on the number of changes perceived during an interval.”*
Piaget (1927/1969)	“*…preoperational children have a notion of time that is tied to events; children's concept of temporal duration is not perceived but constructed based on inferential processes.”*
Ornstein (1969)	“*…remembered durations lengthen as a function of the number or the complexity of events during encoding.”*
Block and Reed (1978) and Block (1985)	“*…retrospective duration linearly increases as a function of the amount of contextual changes remembered in a given time period.”*
Poynter (1983; 1989)	“*…retrospective duration is a function of the number, the discreteness, and the salience of events in memory.”*
Roseboom et al. (2019)	“*…we build an artificial neural system centered on a feed-forward image classification network, functionally similar to human visual processing. In this system, input videos of natural scenes drive changes in network activation, and the accumulation of salient changes in activation are used to estimate duration. Estimates produced by this system match human reports made about the same videos, replicating key qualitative biases, including differentiating between scenes of walking around a busy city or sitting in a cafe or office. Our approach provides a working model of duration perception from stimulus to estimation and presents a new direction for examining the foundations of this central aspect of human experience.”*

Time is a fundamental concept and ubiquitous dimension humans use to coordinate their actions and organize their memories. With the acquisition of the concept of absolute time, we relate to it as the ultimate dimension of our coexistence and shared experience. Unlike its counterpart, “space,” which has extensively been studied in animals and is readily available from local sensory input (proprioceptive, visual, olfactory, and auditory) as a result of locomotion and other spatial behaviors (e.g., “*animal navigating through path integration or positioning of spatial landmarks on the navigation path”*), “time” is not directly observable (van Wassenhove, 2023, p. 4). There is no dedicated sensory input, i.e., no specific receptors or organ in charge of temporal sensation, unlike those for sight, hearing, smell, and touch (e.g., Wittmann, 2009; Merchant et al., 2013).

Nevertheless, no doubt one can experience the passage of time through its indirect consequences (i.e., progressions or regressions) ranging from a very personal (e.g., aging) to more externalized (e.g., change of the season) or from micro time scale (e.g., perceptually undetectable or very brief occurrences) to macro time scales (events lasting months, years, decades or longer, often only graspable narratively). While the passage of time is not directly detected by any sensory system, change is a constant and ever-present source of temporal information. However, the question arises: how do we abstract the temporal dimension when all our sensory inputs report changes simultaneously, either due to the dynamic and fluctuating environment or our own behavior?

Our experience of change provides us with at least two key concepts: succession and duration (for an elaborate take on the taxonomy of temporal experiences, see Pöppel, 1978). Succession pertains to the sequence of events, i.e., the ability to perceive that two or more events are organized in an ordered sequence along the temporal dimension (Fraisse, 1984), such as a causal chain of events. Duration is the time elapsed between two events or the duration of the event itself, meaning that if there is no event, there is no duration either (Fraisse, 1984). Similarly, Gibson (1975) stated, “Events are perceivable, but time is not” (in Fraisse, 1984, p. 2), emphasizing that time is perceivable only through the events since time itself cannot be sensed. Human perception of time is flexible and can change based on our emotional or cognitive context (Nobre and O'Reilly, 2004; Droit-Volet et al., 2007; Eagleman, 2008; Wittmann and Paulus, 2008, as cited in Koch et al., 2009).

Instead of relying on conventional systems (e.g., hours, months, or years), humans are more often inclined toward discrete time experiences. Everything on Earth, including living organisms, is in constant motion or evolution; hence, quantifying an ever-changing “environment” can serve as an expression of temporal experience. The quotation “*We do not remember the days; we remember moments”* (Pavese, 1980, p. 172) illustrates well how mental representation of events holds the capacity to transform continuous physical reality into more discrete phenomenology.

The way humans perceive the order of events, i.e., the conscious chronology, is influenced by both non-linear neurobiological systems (from the level of single neurons to the system level) and active self-regulation (cognitive processes) (van Wassenhove, 2023). In order to comprehend time, the non-linear nature of time is transformed into a linear structure using topological mapping, where, via coding and tagging, every event can be represented on one's life map (van Wassenhove, 2023). Once the external occurrences are tagged as such, their mental representations are susceptible to memory manipulations, retrieval, and reconstruction processes.

To define further what constitutes an event, one may consider its duration and timescale. Events can be as short as hundreds of milliseconds (Michotte, 1963), encompassing discrete moments of interaction between objects, such as a collision. Nevertheless, events can last for extended periods of time, such as life events, like birth, or historic events, like World War II, or cosmic events, such as the formation of the solar system, which took about 600 million years (Teigen et al., 2017). Considering the disparity between the broad time scale of events and the potential underlying neuronal mechanisms that discriminate between them, one should be concerned with the shortest duration at which perception can discern the succession of moments from a single moment, such as the flicked-fusion frequency that can discern the frames of animation from a continuous sensation of motion.

Although there is no agreed-upon definition of all the specificities of these segments (for a thorough discussion on *moments-events-boundaries-periods* and on what constitutes an “event”, see Yates et al., 2023), the idea that experience can be broken down into events is a fundamental concept in cognitive science, and as such, heavily preserved and present among all nuances of event cognition's theories. To be perceived as distinct and not as simultaneously appearing occurrences, events must be separated by a minimal temporal interval of (~200 ms), in which the duration amount differs for the different senses (hence the term “fusion threshold”) (Pöppel, 1978). The critical fusion frequency for human subjects is 30–40 Hz (Eisen-Enosh et al., 2017). That is when the sense of continuity is replaced by a discrete flickering sensation. However, the flicker sequence does not dissociate to independent events in time until the temporal gap exceeds 200 ms (5 Hz with repetitive stimulus) (Wertheimer, 1912; Sekuler, 1996; Ekroll et al., 2008).

Moreover, different modalities resolve the temporal sequence of events at different scales. A well-documented finding in psychophysics is that the auditory system has better temporal acuity than visual (Penney and Tourret, 2005; van Wassenhove, 2009; Merchant et al., 2015, as cited in Rammsayer et al., 2015). However, to determine which one of the events appeared first or second, the minimal inter-stimulus-interval between two successive stimuli must be about 20–40 ms, irrespective of sensory modality (Hirsh and Sherrick, 1961). “For very short stimuli, neither a beginning nor an end is experienced, and thus no experience of duration is provided” (Rubin, 1935, as cited in Pöppel, p. 715).

Whether our brain actively segments the continuous flow of sensory input into meaningful chunks (precursors of episodes) or the sensory input is sampled in discrete snapshots and subsequently interpolated by dedicated areas, such as the motion-processing areas of the brain, is not at all certain. The described sampling most probably stems from visual information processing, as supported by the findings of patients suffering from akinetopsia (i.e., motion blindness) and being unable to experience visual motion. This rare neurological disease involves the bilateral focal lesion of the middle temporal area V5/MT as part of the dorsal stream of visual processing, leading to the complete inability to perceive continuous motion in the environment (for a review, see Zihl and Heywood, 2015). Taking this evidence by its face value, it suggests that without the integrity of cortical area V5, the rest of the brain processes sparse-sampled “snapshot-like” input from the sensory afferents 2–4 times per second. Hence, the sensory input may already be organized according to discrete snapshots. However, these moments do not represent the critical constituents of episodic memory as defined by Tulving (below).

## 2 Why do events matter?

### 2.1 Cultural anthropological perspective

From the perspective of adult contemporary humans in industrial societies, it is almost unimaginable to take the stance and “navigate through” time without clock-based metrics; however, that is precisely what our ancestors were compelled to do many years ago. As frequently reported in various anthropological studies, humans relied on the diurnal and seasonal natural cycles before being introduced to the mechanical clock-time and standardized time units, and generally, a metric system, as the most rudimentary time tracking forms. Along with the rotation of the celestial bodies, almost as naïve statisticians, humans exploited the temporal regularities and the oscillations of the internal biological clocks (e.g., one of the “Ishango bones”—a baboon's fibula bone found with the incisions that are speculated to correspond to a menstrual cycle tracker, found in the coastal areas of the Democratic Republic of Congo, dating from the Upper Paleolithic Period, ~20 000–25 000 years ago), and later on, with the advanced development of civilization, social norms, and conventions were added to generate a sense of duration and provide structure to the non-metric time (Silva Sinha, 2019). In conclusion, the event-based time interval terms were used to refer to the interval either as a reference point or landmark in time or to the duration of the interval (Silva Sinha, 2019).

Even though such a concept of time vastly differs from the “clock time” used nowadays in Western culture, there are some “event-time” remnants. In isolated cultures of the Amondawa Amazonian tribe or other indigenous populations (Huni Kuĩ, Awetý, and Kamaiurá, as investigated by Silva Sinha, 2019) where such exclusively event-based time concepts are still enabling successful communication and mutual alignment, even in the absence any advanced timekeeping devices and refined time tracking systems as calendars (more detailed in Levine, 1997; Sinha et al., 2011; Brdar et al., 2020).

Although rare, there are still instances where event-based time measurement is used in modern Western and non-Western societies. These cases are minimal and usually involve specific activities. For instance, while cooking, instead of assigning each step of preparation a metric value and following the strict temporal timeline, recipes are described in terms of the result to be achieved and rely dominantly on the event description, e.g., “the other ingredient should be added after 4 minutes” versus “the other ingredient should be added when the water boils”. This type of event-based time measurement is achieved through image schemas, prepositions, and pseudo-sublative, as explained by Brdar et al. (2020).

### 2.2 Ontogenetical developmental trajectories: from event-dependent to event-independent

Apart from the *cultural-anthropological* perspective, similar shifts can also be observed at individual levels. Namely, before being introduced to conventional time or transitioning to the “clock time” type of metric to infer the temporal duration, children primarily rely on heuristics indirectly related to time, such as event density.

Before time is processed as a separate dimension, it is computed with or inferred from other metrics. Children at a very young age can distinguish between various magnitudes, including “big” and “little,” or tell whether something is “short” or “long” (Ravn and Gelman, 1984). According to the Theory of Magnitude (ATOM, Walsh, 2003), space, time, and number (i.e., size, duration, and quantity) are all processed by a single, innate magnitude processing system. Due to the innateness, the ability to differentiate magnitudes is readily available early on and becomes more accurate and refined as children age (e.g., Droit-Volet et al., 2008; Halberda and Feigenson, 2008; Odic et al., 2013). ATOM is not exclusive among other theories of time emerging later in life, e.g., Mental Timeline (Bonato et al., 2012; Bender and Beller, 2014; Magnani and Musetti, 2017), which advocates the cultural inclinations in magnitude representation rather than the innateness of magnitudes, as ATOM.

The next question is how the initial temporal metric is computed while dependent on the shared magnitude processing system, i.e., when the shared magnitude processing system is the only metric available. For instance, in a conversation with a 4-year-old on how much time is left until Christmas, the abstract concept of calendar months or any other time units will do no favor. Therefore, we often convert the question to more concrete concepts, such as sleep, and answer the question, “*How many times do we need to sleep until Christmas?”* Here, a few nights would span a conceivable interval for a 4-year-old to maintain their anticipation instead of many nights that would be inconceivable and hence would not endure their anticipation. In other words, we quantize longer durations by events, and then the frequency/density of the events is utilized by the notion of duration.

Another example of a quantity being converted to duration (and used interchangeably) on a daily basis is when children are gesturing with pointed fingers to address their own or someone else's age. This magnitude objectification, even without a fundamental understanding of time or numbers, is metonymically linked with the age where “more fingers” means “more age” and where “four fingers” are more than “two fingers.” Such “event time” depends on the events' density—their frequency and succession; the more likely an event is available^1^ from memory, the longer the duration it represents. Likely, there are shared neural responses between numerosity and time, as has been demonstrated by recent research work using an fMRI (e.g., Hayashi et al., 2013; Fortunato et al., 2023).

Moreover, temporal representations heavily rely on spatial representations: Piaget extensively studied, and was the first to provide experimental evidence, that children's concept of time is not adequately differentiated from their notion of space during the initial developmental stages, suggesting both symmetric (“time and space form an inseparable whole,” 1927/1969, p. 1) and asymmetric relationships (“in the case of space we can ignore time…”; “when it comes to time we cannot abstract the spatial and kinetic relationships,” (p. 2). In his later works (e.g., 1946/1970), Piaget implied that children often rely on spatial cues to make temporal judgments. However, a series of empirical findings disentangling the relationship between spatial and temporal representation (e.g., Casasanto and Boroditsky, 2008; Casasanto et al., 2010) has confirmed that spatial judgments are viable without temporal cues. Despite the asymmetry in this time-space interdependency, the smooth conversion between spatial and temporal domains has become the core of the Metaphor Theory (Lakoff and Johnson, 1980/2003). Due to the stated overlapping of the domains, representations of space lay the foundations for representations of time, and despite the initial separable dimension of duration information, representations of space ultimately become the fundamental building blocks for time (Casasanto et al., 2010).

Time's nonindependence from space has deep linguistic footprints. The early undifferentiated phase of time and space and their interchangeability are reflected in many languages as a spatialization of temporal concepts such as “length of time”, “range of time”, and “time span”. The opposite when a spatial concept is expressed by a temporal relationship is also evident in examples such as the “beginning and end of the hallway”. Here, the “beginning” and “end” are temporal expressions applied to depict spatial relations. This cross-over between the spatial and temporal expressions is consistent with the early undifferentiated concept of the spatial and temporal dimension of pre-kindergartener children, as originally observed by Piaget (1927/1969).

**Figure 1:**
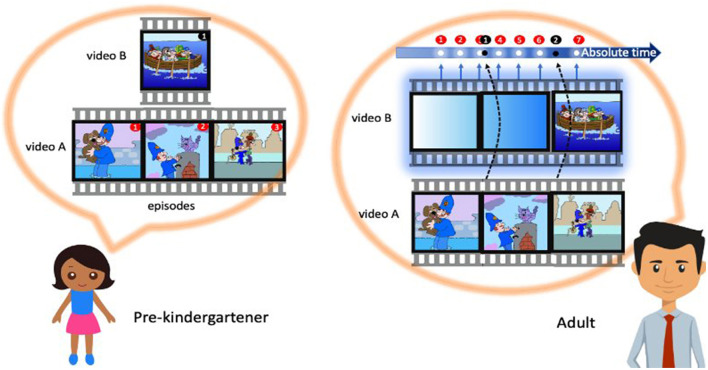
Use of sampling and availability heuristics in temporal cognition. Do both children and adults rely on the events as units to construct a subjective passage of time? A recent study by Stojić et al. (2023) found that children and adults perceive time differently based on the content or the density of events^a^, i.e., while children focus on the number of events, the adults pay attention to the gaps between them, i.e., the so-called event boundaries. In a screening of the eventful (action-packed material made of several segments with defined “beginning” and “ending” and narratable content) and eventless cartoons (monotonous and repetitive actions with a single episode and, therefore, no storyline) despite objectively equal duration and a balanced number of characters as well as moving elements, different age groups claimed cartoons as different in durations. Such finding was attributed to different decision-making processes in children and adults–children use the availability heuristic, or the “how much they can talk about something” rule, and adults rely on the sampling heuristic, or the “how many times they were able to sample the flow of an absolute time” rule. ^a^Considering the lack of a universal definition, in this context, the events are meant to be narratable, with a distinctive beginning and ending, following Zacks and Tversky's (2001) event segmentation theory.

The ability to mentally travel through time is facilitated by the human capacity for language, music, thinking, and generally, the operations that are, in principle, abstract and, therefore, whose temporal structure is not fully understood and extremely difficult to characterize (van Wassenhove, 2023). Parallel with the accompanying milestones occurring in development (e.g., use of metaphors as linguistic figures or expansion of the general vocabulary enabling the expression of various fractions of time), the concept of “event time” is replaced with the so-called “clock time”, that is characterized as *abstract, externalized, linear, unidirectional*, and *more mature* (McCormack, 2015). The development of time concepts follows a trajectory similar to spatial cognition: from egocentric to allocentric, from context-dependent to context-independent, and from incidental to absolute. We can see the evidence for a leap from the event-density-based metric of elapsed time to duration estimates based on sampling heuristics (Stojić et al., 2023) (Figure 1). According to the sampling heuristics applied to duration estimates, the time is absolute and external, consistent with the notion of Newtonian time. Then, experiencing the flow of time is equivalent to the procedure of sampling it. Sampling can be done by checking the time on a clock or simply by being aware of it or paying attention to it and adding up the moments of this “time awareness” together. We also know that the shift from one representation system to another occurs around the age of 5 (McCormack and Hoerl, 2017), while the change in underlying decision-making models (i.e., from availability to sampling heuristics) occurs between the ages of 4 and 10 (Stojić et al., 2023).

## 3 The supporting role of episodic memory

In retrospective amnesia, the memory of the particular events, the *time tags* of the events, i.e., the time stamps when exactly they occurred on the autobiographical timeline (or in a temporal dimension if the events are not personal) are lost, and the metacognitive computation of the relative distances of two or more events, or a particular event to the moment of *now*, is not feasible. Patients suffering from Korsakoff syndrome lose the *time tags* of the events while the memory of the event remains preserved; hence, they can retrieve the event itself but are unable to determine its sequence (van der Horst, 1932, as cited in Pöppel). Episodic memory is an integral part of any retrospective demand (e.g., Hicks et al., 1976; Block, 1985), and different mechanisms, as per neuropsychological studies, are employed in different tasks and operations. Humans rely on their memory for the number and coherence of contextual changes to estimate time retrospectively (Bangert et al., 2019). A review of the developmental aspect of episodic memory is needed to elucidate how the events organize themselves to support memory function. Per definition, episodic memory encodes events by their spatial and temporal coordinates for both short-term and long-term conscious recollection (Tulving, 1972). The episodic memory, as an information processing system, has three functions: (a) receiving and storing information about events or episodes that are temporally dated, along with the temporal and spatial relations among these events; (b) retaining various aspects of this information; and (c) transmitting specific retained information to other systems, including those that are responsible for translating it into behavior and conscious awareness (Tulving, 1972). According to this notion, retrieval of episodic memories relies on the re-instantiation of contextual markers present during encoding, such as spatial cues and preceding or subsequent events. An inherent feature of this spatiotemporal fabric is the temporal order of events unfolding (Kahana, 1996; Howard and Kahana, 1999). The temporal structure of these events accounts for the concepts such as simultaneity, irreversibility, and temporal order.

Although we cannot further assert how those concepts develop in children's brains, we can pinpoint their macroscopic anatomical domain with certainty. Episodic memory is critically dependent on the integrity of the hippocampus and associated medial temporal lobe structures (Scoville and Milner, 1957). The hippocampus, for instance, undergoes a 15% volume increase between the ages of 2 and 6 (Reinhardt et al., 2020), more than twice as fast as cortical thickness increases (~6%) over the same period (Gilmore et al., 2018). The rapid volume increase of the head of a hippocampus is not explained by the isotropic increase of brain volume, and moreover, the volume increase in the head of a hippocampus is not isometric to other subregions either (e.g., body or tail). These developmental differences in hippocampal subregions might underlie the developing course of episodic memory during early childhood (e.g., Jabès and Nelson, 2015; Lavenex and Lavenex, 2013; Riggins, 2012; Serres, 2001, as cited in Riggins et al., 2016). Several behavioral studies consistently identified a rapid improvement in episodic memory abilities between the ages of 4 and 6 (Bauer, 1996; Drummey and Newcombe, 2005; Sluzenski et al., 2006; Riggins et al., 2015, as cited in Riggins et al., 2016). Riggins et al. (2015) found positive correlations between episodic memory and the volume of the hippocampal head bilaterally in 6-year-old children but not in 4-year-old children. A year later, the same researchers found that the hippocampal functional connectivity of 4-year-olds and 6-year-olds is generally similar; however, while hippocampal functions become progressively integrated with the cortical memory networks with age and segregating from regions unrelated to memory, episodic memory ability rapidly improves and attains adult-like connectivity patterns as early as 6 (Riggins et al., 2016).

From the evolutionary perspective, rather than remembering or perpetually reminiscence about the past, the purpose of memory is to predict and prepare for future events, i.e., “to allow animals to anticipate (i) what will happen, (ii) when will it happen, and how to respond to it when it happens” (Buonomano, 2018, p. 18). The ability to anticipate what is about to arrive is one of the fundamental elements of cognition; Lashley (1951) and Mach (1885), among others, argued that the rhythmic mechanisms are necessary for the organization of our behavior. Likewise, Pavlov, famously demonstrated the critical role of rhythmic phenomena in establishing conditioned reflexes via associate learning in the experiment with dogs when the animals learned the duration of the latency between the onset of the sound of the bell and the delivery of the food. In other words, if the occurrences that are regular in their patterns of appearance are successfully coded and memorized, the extrapolation is feasible, allowing for anticipation and preparation of various kinds. The described ability to predict is enabled through internal mapping of the events in time, termed “phase sense” (Gallistel, 1990). The mental record of our past experiences is a crucial part of the “self,” and the continuity of the “self” provides us with the most reliable sense of reference, i.e., a viewpoint, to understand the world around us.

Episodic memory, however, is not limited to being mere mental representations of specific past events; they are complex and multi-dimensional information that encapsulates not only the details of a particular experience but also the emotions, sensations, and context surrounding it (Mahr and Csibra, 2018). Mahr and Csibra (2018) argued that episodic memory should be recognized as a distinct epistemic attitude toward an event simulation due to its meta-representational format, i.e., a unique way of representing past events. For instance, the anatomy of one single episode can often be described as the “Who did what, where, and when?” Hence, when we learn new stories, they typically involve characters who are either enabling, causing, or intervening in some events that have consequences. The outcome of these actions or interactions often entails an event that triggers a new event as part of a new episode. Children start parsing episodes^2^ according to actors and actions and causes and effects earlier than they acquire the necessary vocabulary to describe their actions and motivations (Johnson et al., 2008). That said, somewhere between 4 and 6 years of age, episodic memory emerges (e.g., Perner and Ruffman, 1995; Perner, 2001; Tulving, 2005, as cited in Chen et al., 2013). Simultaneously, events become building blocks of episodic memory, and they remain fundamental constituents of autobiographic memory throughout the lifetime (e.g., Bauer et al., 2012; Drummey and Newcombe, 2005; Sluzenski et al., 2006, as cited in Riggins et al., 2016).

By possessing a dedicated mechanism to manage claims of epistemic authority effectively, humans are able to ensure that the information they rely on to build their knowledge is accurate, thorough, and reliable. As such, episodic memory is essential to maintaining both one's integrity and understanding of history via autonoetic consciousness (Tulving, 1983). Conveying the testimonies of others encompasses the broader context and social relevance, i.e., the coordination of social realities with others (Mahr and Csibra, 2020) that is built by the noetic consciousness, which is factual and non-intimate, relative to autonoetic.

From the phenomenological view, episodes also have their temporal reference frames defined relative to virtual observers, just as in the domain of spatial cognition. When the temporal structure of episodes represents events from an observer's present-time point of view, events are assigned to the past, present, or future. This view represents an egocentric perspective centered on the observer's present time. However, events can be considered as preceding or following a specific event in focus, for instance, “*the events before the pandemic…”* or “*the events following the collapse of the stock market …*”. These would be examples of event-centered perspective-taking or event-focused episodes. Events can also be arranged relative to the time axis of absolute time, such as historical references or composing a curriculum vitae. We take an objective distance from the subject, such as our life, or in the case of writing a curriculum vitae, we tag the events by the years of occurrences in absolute time. This type of allocentric perspective taking on time only manifests later in adolescence, most likely when personal life has become a narrative. By late adolescence, developing a mental timeline and, in parallel, reorganizing old and recent memories according to that mental timeline, the time dimension of autobiographical memory, is crucial. Adults arrange their memories according to the order they lived them through, and children follow the same pattern, which makes the representation of the past an active and constructive process (Fraisse, 1964, as cited in McCormack, 2015). However, a sole listing of the events will not constitute the concept of the past without the “self” playing a role as the common denominator between temporal consciousness and memory processes, as pointed out by Povinelli (2001; Povinelli et al., 1996, as cited in McCormack, 2015). That is, the memories of the “self” in the various stages of occurrences that already happened allow one to fit the self onto the temporal timeline and all the different extensions of the self. Reflecting on how memories and perspectives on events are interconnected, which is often attended to in interactions with parents, helps one position oneself in a broader time perspective and comprehend cause and effect, which ultimately underlies the concept of linear time (McCormack, 2015).

## 4 Conceptualization of the temporal order of the events

### 4.1 Causality and distance between events matter

How do children learn to locate the time and generate *time tags* before they adopt clocks and calendars, as standard examples of non-perspective temporal frameworks? To remember the sequences of events they are familiar with, children, as young as three, construct the representations of events and describe the sequence of how the events typically unfold (McCormack, 2015). Although they are not temporal frameworks *per se*, these so-called scripts, schemas, or generalized event representations are the first mechanisms used to distinguish “before” and “after” and build the ordinal sequence along the timeline (McCabe and Peterson, 1991; Nelson and Gruendel, 1986; as cited in McCormack, 2015). As toddlers transition to preschoolers and their memory capacities increase, they can remember longer sequences of information and retain them for a more extended period of time (see Bauer, 1996; Hayne, 2004, according to McCormack, 2015).

Scripts are minimal, but they capture two critical attributes of the flow of events that will be integrated into the concept of causality: (i) sequential order (irreversibility) and (ii) normative nature. Scripts, for instance, “it is bedtime after the teeth are brushed” or “we put on our shoes, and then we leave the house”, are rudimentary and devoid of any causal explanations (McCormack, 2015). They are adopted as generalizations of recurring sequences of actions (e.g., one-always-leaves-the-house-after-the-boots-are-on) with limited flexibility to adapt them to different thematic contexts. Another limiting feature is that events organized in a certain sequence are allocentric, i.e., only ordered relative to each other, without referencing the person observing or interacting. This lack of reference is reflected in the indifferent linguistic constellation, or as Nelson and Gruendel (1981) and McCormack (2015) pointed out, “children would describe such sequences in the second person using the timeless present tense” (McCormack, 2015, p. 22), such as “You have a bath, and then you put on your pajamas” (Nelson and Gruendel, 1981, as cited in McCormack, 2015, p. 22). Scripts enable the child to represent the sequence of events but do not assign specific time annotations to them—tonight's bedtime sequence is no different from the sequence from the night before, etc. Conclusively, while preschoolers can conceptualize that most scripts are repeatable (a defining feature of scripts) and ordered (irreversible), it is incorrect to assume the concept of tenses at that age. That is why Friedman referred to scripts and any memorized time locations that have no value of relative times but operate on the association basis as “islands of time” (Friedman, 1992, p. 186). Nevertheless, these mechanisms are essential for children to make a correspondence between the sequence of events and the stream of physical reality and provide a framework for children to orient in time. Needless to say, the correspondence is hardly isomorphic due to the distorting effects of attention, emotion, and memory processes (e.g., van Wassenhove, 2023).

As scripts are used to denote the temporal order of the events that are closer in time, i.e., events happening right after each other, that can leave one questioning what kind of mechanism is underlying the representation of the events that are not temporally merged. In other words, scripts, although nontemporal in their essence, could be a precursor to temporal cognition, but what follows after the scripts? How does the “event concept” precede the “day concept,” and how are the events that are not related to each other represented? How are those learned chunks of sequences connected, and does that happen before adopting the unified temporal frameworks, such as clocks and calendars?

Between causality and events, there is an intriguing connection: events closer in space and time are more likely to be perceived as causally related than events further apart (Hume, 1748, as cited in Muller and Nobre, 2014). Similarly, per the Bayesian causal-binding explanation, causal beliefs can lead to temporal compression. Temporal compression can occur simply by perceiving causality, even if it is not actually present (Buehner and Humphreys, 2009), and temporal compression is more robust for action–effect pairings that are closer in time (Haggard et al., 2002, as cited in Muller and Nobre, 2014).

Interestingly, for children under the age of 4, time is ordinal. Time only represents an episode as a succession of events without precise time tags attached to the events. For instance, a 4-year-old can understand that she will only get ice cream after lunch but cannot assert whether lunch will take 10 or 20 min. Later on, these intervals will be associated with specific durations, and the child may be able to assert that the ice cream truck will be gone by the time they finish lunch. Hence, the ordinal scale of events is replaced by a scalar scale, where events can be arranged in absolute time and durations, like in a Gantt chart.

Although several lines of research from the cognitive development field suggest that children are capable of perceiving both duration and succession at a very early age, it is not until around the age of 7 or 8 that children develop the ability to think logically and understand the coexistence of these two concepts (Fraisse, 1984). According to Friedman, the onset of the abstract notion of time coincides with the emergence of the mentioned ability and gradually develops from this age onwards (Friedman, 1982, as cited in Fraisse, 1984).

Similarly, as the increased memory capacity allows for generating the narrative via the accumulation of scripts, increased memory capacities play a crucial role in the causation by using the events to represent the causal structure of the episodes. As previously elaborated, a single episode can be broken down into the key elements of “who did what, where, and when?” New stories involve agents performing actions with consequences, leading to events that trigger a new event as part of a new episode, almost like a chain reaction. Children learn this structure early on and describe their own actions and motivations when recounting stories. During the preschool year, children are able to produce “coherent narratives of their personal past, with a well-defined causal structure,” as documented in studies conducted by McCabe and Peterson (1991) and Fivush et al. (1995), as cited in McCormack (2015). Gradually, those temporally structured event representations gain the flexibility to transcend the circadian limit. Supra- and super-circadian range should matter, and the daily intersection might be critical due to the discontinuous nature of consciousness (e.g., Baars, 1988) (for an elaborated view on continuity and discreteness of consciousness across the stages of wakefulness and sleep, see Horton, 2017, as on the problems of the dividing the concepts of consciousness to continuous and discrete, see Hayes and Hofmann, 2023).

Based on this approach, we assume that: (a) the interchange of the sleep-wake cycle and the shifts in a state of consciousness represents a significant shift in the internal introspective milieu. In other words, not only does the sleep-wake cycle interrupt the conscious stream within which the events take place, but it organically isolates events under different segments of conscious streams. Consistently, it has been demonstrated that the brain receives reduced internal and external environmental inputs (Steriade et al., 1993) and exhibits a different oscillatory pattern during the sleep and awake periods, consistent with acquiring information and consolidating memories (Buzsáki, 2006). The lower level of consciousness during sleep results in the complete loss or altered spatial and temporal awareness (but also in disorientation after deep or long periods of sleep or drastic disorientation after longer discontinuities such as being in a coma); (b) sleep itself could constitute an independent event that separates awake epochs, with the conscious state providing a framework within which all the events are happening like in Cartesian theater-like spatial fashion (per Dennett, 1991).

Children are able to establish the temporal causal structure predominantly while conversing with the adults, whose role is often to provide the context and the root of the causality. Let's imagine a child and a mother chatting about a day spent at kindergarten. A child might convey the content partly or inaccurately and make a wrong interpretation or the order of the events as they unfolded, which the older interlocutor is expected to correct. Such scenarios are repeated and rehearsed until children are able to construct meaningful and correct narratives all by themselves, which happens gradually into childhood, positing quality social interaction as one of the means for understanding temporal concepts, especially before the concepts of clock and calendar are acquired (Nelson, 1996).

### 4.2 The direction of an emerging mental timeline

Although time travel does not contradict the laws of physics (see “The Twin Paradox” Einstein, 1920) or adherents of *eternalism* (for further discussion on *presentism* and *eternalism*, see McTaggart, 1908, 1922; Buonomano, 2018), experientially speaking, it is not possible to go back in time or re-do/live certain events/moments except for re-visiting them in memory. In physics, the unidirectional nature of time is associated with the concept of large-scale increase of entropy. Specifically, the second law of thermodynamics postulates that a disordered macroscopic state is less likely to evolve to a less disordered state than a more disordered one. Hence, going back in time is not impossible but rather improbable. The aforementioned is consistent with the perceived unidirectionality of events unfolding around us, such as the fragments of a shattered glass vase that are unlikely, but theoretically, not impossible, to reassemble themselves to the shape of a vase (unless, of course, by human effort). Because of that, the abstract notion of absolute time, as derived from our experience and interaction with the world, is irreversible and unidirectional.

From a cognitive perspective, the preference to organize and align memory and actions along a line can be understood as a dimensionality reduction. A one-dimensional timeline can effectively reduce the complexity of the world in which multiple objects interact simultaneously, and the configuration evolves from one state to another, such as in the example of the shattered glass vase. If we track the trajectories of each individual fragment separately from others, that will end up as a high-dimensional representation. In contrast, by representing all the fragments on a shared timeline and assuming that all the trajectories were simultaneously evolving, we reduce the number of dimensions and isolate time as the common underlying dimension that can organize every event.

Such linear representations of time are also preferred because of their minimizing effect on visual processing. That is, when time is represented in the form of straight lines or successive order, the sequences of events have the smallest possible distances between the consecutive points, which further saves the overall time and effort required by visual processing (Tillman et al., 2022). Likewise, as linearly sorting the temporal sequences eases the demands on visual processing in the present-or close to present tense, representing the past chronologically might facilitate the reconstructive actions inside the memory domain.

Besides the “minimal effort” hypothesis, there is strong evidence from the developmental studies according to which the human brain's hemispheric asymmetry in terms of the cortical volume, and hence, the functionality, favors the linear representation and left-to-right direction (e.g., de Hevia et al., 2014). In human infants, the right hemisphere functionally matures faster than the left during prenatal and postnatal life (Rosen et al., 1987; Tzourio-Mazoyer et al., 2002, as cited in de Hevia et al., 2014). The cortical asymmetry originates from the right hemispheric specialization for visuospatial processing (Mesulam, 1981; Vallar, 1998; de Schotten et al., 2011; as cited in de Hevia et al., 2014), i.e., the right hemispheric dominance during the visuospatial tasks, which results in a leftward hemifield bias. Apart from humans, the bias to attend to the left side of space has been shared with non-human species (e.g., chicken, Rugani et al., 2010, 2014, and fish, Dadda et al., 2009), strongly suggesting that cultural inventions (e.g., numbers) and educational effects (e.g., orthography, directional finger counting, directional object counting preferences) might not be the isolated determinants when it comes to oriented spatialization of numbers.

Although a tendency to represent time as a line can be argued as an oversimplification, it seems that the direction of the timeline is not universal and that different cultures have varying directions for their timeline representation. In other words, when spatially representing the sequence of temporal events, native English speakers arranged time sequences of events following the left-to-right fashion. The same seemed to be the case with several other languages, with the congruent direction of writing and speaking (e.g., English, French, Spanish, Dutch), i.e., left-to-right orthography (Casasanto and Boroditsky, 2008). In contrast, Hebrew and Arabic speakers followed the right-to-left orientation, again being consistent with the direction of reading and writing of the spoken language (Fuhrman and Boroditsky, 2010). Likewise, when asked to express the time spatially, English speakers tended to map time horizontally, i.e., from left to right, while Mandarin speakers described time vertically (from top to bottom) (Boroditsky (2001; 2011; Bergen and Lau, 2012). Native Croatian speakers at kindergarten age used horizontal and vertical gestures in the 50:50 ratio, while the ratio increased to 85% among 8–10-year-olds in favor of horizontal hand spreads (Stojić et al., 2023). Thus, although the conceptualization of time in terms of space appears to be universal, the following led to the conclusion that the direction in which time passes is culturally determined and strongly related to the direction of reading and writing of a specific language (Autry et al., 2020).

### 4.3 Past or future, first?

**Figure 2:**
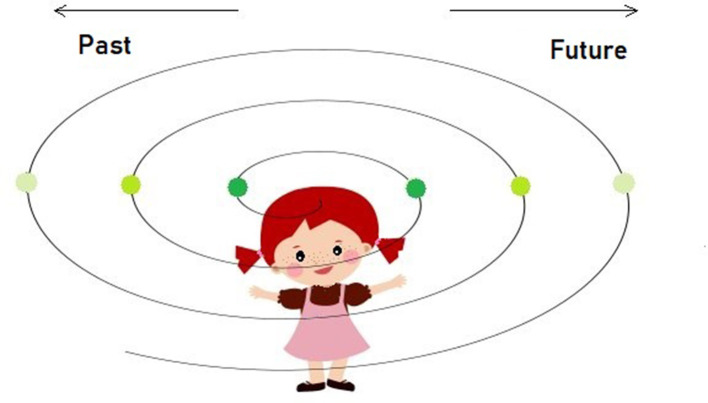
Illustration of the bi-directional model where the same-colored points in space-time represent the equidistant locations relative to self, further suggesting they are both acquired at/around the same time/developmental stage irrespective of the direction—past or future. Our bi-directional model adheres to the undifferentiated stage when time is egocentric and not yet unidirectional. We proposed that this undifferentiated time/space concept only lasts until the child replaces it with the more differentiated Newtonian concept of absolute time and space around school age. Before that transition happens, children between 4 and 6 often resort to the sequence of events to intuit duration when they have to, expressed by event density as an estimate of time.

In a quest to dissociate the two fundamental dimensions of the “timeline” concept: (a) the sequential order of events (ordinal variable) and (b) the difference between past and future (binary variable), the question would be, which one develops first? Here, we consider the possibility that the child's knowledge expands bi-directionally relative to its position on a spacetime continuum (Figure 2). It means that children acquire the knowledge or, better said, the sense of a distance between “tomorrow” and “yesterday” (or any other equidistant points in the past or future) in relation to “now” at the same time and then gradually progressing to more distant time allocations. The distance from the “now” moment, heading either to the past or future, is dependent on the memory and mentalization capacities, meaning that, as the storages expand, it is likely to reach far distance timepoints and, likewise, to imagine them. This sort of egocentric perspective could also be explained by the lack of the time flow direction that is culturally acquired and predominantly linear (e.g., from left to right for Western-speaking nations, Boroditsky, 2001), along with the lack of the concept of entropy or irreversibility among the young children.

Further, on timeline direction, it is common for pre-kindergarteners to omit the correct order of the events on the timeline and to interchangeably use the events from the past, relocating them in the future, and vice versa, to speak of the prospective events as they already happened (e.g*., When I was a grown-up, I used to spend all summer at my grandma's place*). The border between the past and future seems fuzzy in their narratives. That children can still be erroneous in this conceptual matter even until the age 8, i.e., “confusing the near future with the recent past” (Friedman, 2000, p. 1), has been noted in the work of Friedman and Kemp (1998) and Friedman (2000); (2002). Such anecdotal and, nevertheless, frequent cases could reflect a limited speaking vocabulary (i.e., lack of appropriate and extensive use of adverbs and prepositions). However, they could also easily count as a supporting argument to the above proposed bi-directional trajectory in the making.

Although this bi-directionality assumption still lacks firm experimental support, it complies with one of the most fundamental principles of psychophysics, the Weber-Fechner law, as cited in Brietzke and Meyer (2021);

“*Weber–Fechner law refers to the observation that across perceptual domains (vision, hearing, taste, touch, and smell), physical changes in stimuli are logarithmically compressed in perception such that the farther they are from an original stimulus, the less well people differentiate between them” (Fechner*, *1948**) Specifically, in the cognitive sciences, “compressed representation” refers to the phenomenon in which representations do not show the same degree of acuity for all parts of the scale on which they are measured, with later ends of the scale harder to tell apart (i.e., “compressed”) than earlier ends of the scale (Fechner*, *1948**; Howard*, *2018**). In simple words, as objects become distant, they also become less discriminable or compressed*.

The account of bi-directionality was probed by Friedman, who was the first to prove that 4-year-olds are able to judge the relative distances of unrelated events, i.e., which one of two events from the child's personal past occurred earlier or later in the past) (Friedman, 1991). Soon after, these findings were further confirmed by Friedman et al. (1995) and Friedman and Kemp (1998) using a different experimental paradigm and children up to the age of 6. These experiments revealed that the young children's ability to determine which of two events occurred more recently is significantly influenced by two factors: the temporal distance of the events in the past and the ratio of the distances of the two events (Friedman et al., 1995; Friedman and Kemp, 1998, as cited in McCormack, 2015). These two categories of mapping systems in the brain Gallistel (1990) referred to as “phase sense” for event location and the “interval sense” for the distance between events (i.e., the duration) in time.

Around the age of 5, children start to understand the concept of future time and are able to differentiate between events that will happen in the coming weeks and those that will happen many months away (Friedman, 2000; McCormack and Hanley, 2011, as cited in McCormack, 2015), and by the age of 7, the ability to judge the relative future distances are completely mastered (Friedman, 2002).

## 5 Events and duration

Along with the “order”, another critical feature of subjective time is the duration (Fraisse, 1984). It refers to the amount of time or the interval elapsed between the two successive events or as a difference in phase points (Gallistel, 1990, 5:05). It can vary on micro- and macro scales: from a very brief perceptually undetected or by the episodic memory, encompassed interval, or surpassing one's lifespan in a sense that has to be organized in a collective and cultural timeline, otherwise better known as history. As it is vital for any action or bodily movements, scheduling and attending activities, vehicle management, and synchronization in general, the percept of duration is an integral part of human experience, playing a vital role in everyday behavior and the survival of an individual organism (Pöppel, 1997; Wittmann, 1999; Buhusi and Meck, 2005, according to Wittmann, 2009).

Existing studies have found that computing the durations for prospective (durations starting in the present and ending in the future) and retrospectives (starting in the past and ending either in the past or present) timings seems to involve very different psychological processes (reconstructive cognitive models vs. biological clock-like models) (Zakay and Block, 1997; Tsao et al., 2022).

While the primacy of “order” vs. “duration” in everyday life is arguable, their underlying neural mechanisms are also different. It is known from neuropsychology studies that the skill of keeping track of the order of events, as part of executive functions, continuously improves with the maturation of the prefrontal cortex. The prefrontal cortex starts developing during the early ages but continues into early adulthood (e.g., Fuster, 2002; Gogtay et al., 2004, as cited in Kolk and Rakic, 2022). It is also widely reported that the temporal order is severely impaired in case of prefrontal lobe injuries or any existing anomalies (e.g., Schmitter-Edgecombe and Seelye, 2012; Dulas et al., 2022). Sometimes, patients suffering from Korsakoff syndrome lose the time tags of the events while the memory of the event remains preserved; hence, they can retrieve the event itself but are unable to determine its sequence (van der Horst, 1932, as cited in Pöppel) but that can also be an indirect consequence of general memory deterioration.

Eichenbaum (2013) proposed that the medial temporal lobe is responsible for storing the temporal aspects of memories, encompassing the distance, location, and order of events in a cognitive map (as cited in van Wassenhove, 2023). Studies on rats with hippocampal lesions showed severely impaired retrieving of sequential order of odors, supporting the role of the hippocampus in learning the sequential structure of events (e.g., Fortin et al., 2002). The hippocampus was also implicated in correct sequential recall of past experiences in humans (Lehn et al., 2009) (for a review of the hippocampal-entorhinal region in processing and remembering sequences of events, see Bellmund et al., 2020).

On the other hand, the structures underlying duration judgments (in the scale of fractions of the seconds or longer) have not been identified. The reason why it is challenging to find a consensus on processing and neural models for the subjective sense of duration is simply the implication of several neurophysiological systems from different neurotransmitters involved (e.g., dopamine, serotonin), neurological patients with various brain lesions or neurodegenerative diseases exhibiting severe impairments in temporal judgments, and non-congruent neuroimaging findings, mainly performed with TMS and fMRI.The mentioned is most likely predetermined by different timing mechanisms for different time scales (Trevarthen, 1999; Wittmann, 1999; Mauk and Buonomano, 2004; Buhusi and Meck, 2005, as cited in Wittmann, 2009), including modality-specific processes (e.g., Wittmann, 2009). Another potential reason for perpetually failing to track down the anatomical locations or systems dedicated to duration computation might be the possibility that duration, as a temporal determinant, is not a sensory feature or output product of any of the systems but the brain's constitutive dimension (see van Wassenhove, 2023).

The duration being assessed, sensory modality engaged, and the type of task all govern which timing mechanisms will be employed. More specifically, depending on whether it is about the shorter durations up to one second or longer durations such as hours or even days (and emphasizing the biological or cognitive component in time), some of the most prominent competing theoretical models assume the existence of the accumulator-pacemaker model (e.g., Treisman, 1963; Gibbon et al., 1984, and the attentional-gate model by Zakay and Block, 1997), advocate against the simplistic timekeeping mechanism (e.g., Matell and Meck, 2004; Wackermann and Ehm, 2006; Karmarkar and Buonomano, 2007), or ponder the relevance of the memory processes (Staddon, 2005; Wackermann and Ehm, 2006) (as cited in Wittmann, 2009). Accordingly, the exact anatomical location of temporal processing has yet to be determined. Among other structures, the cerebellum, the right posterior parietal cortex, the right prefrontal cortex, and fronto-striatal circuits have been proposed (cited in Wittmann, 2009) under the assumption that duration tracking exists as a dedicated and not as an intrinsic property of the brain.

Friedman (1993) pointed out that there is no temporal code in human memory, i.e., it is not the duration of the events itself that is being stored. Instead, he asserted that the chronological past is a result of an active reconstruction process, meaning that the human experience of time is a conscious timeline generated by an internal model.

According to Friedman's (1993) terminology, event timing is determined by ordinality, distance of the event from the present, and the location of the event and its accompanying contextual information. In his developmental studies, Friedman has found separate development trajectories for these distinct classifications, such as distance and location judgments (Friedman, 1991). Similarly, Mahr and Csibra (2021) noted that the general knowledge, temporal landmarks, and the content of the event representation are to be held accountable in the post-retrieval stage.

Conclusively, a metacognitive comparison must be performed to determine when the events occurred and how long they lasted on a temporal scale, including the relative distance between them. As part of the autobiographical memory, these memories of either short or long duration or close or distant events obey the laws of recency (i.e., the postulate of the “memory strength theories,” where memory traces progressively decline with the passage of time, e.g., Hinrichs, 1970), and salience, where priority in remembering will be given to a specific characteristic over another (for a general overview of memorability of visual stimulus features and their effect on time perception, see Ma et al., 2024).

## 6 Conclusion

The concept of “events” is crucial to understanding human behavior and temporal cognition, reflecting how humans attend to the surrounding dynamic world (Yates et al., 2023). Temporal cognition revolves around events—event-based time is a central source of orientation during early development, and later in life, event-independent time is a hallmark of a mature processing time, typical for adults. Although there is no agreed-upon definition of the specificities of these segments, primarily due to the intricacies of what constitutes an event, the idea that experience can be broken down into events represents a fundamental concept in cognitive science. As an indispensable step toward perception, event segmentation, i.e., the transformation of continuous physical interaction with the environment into discrete experiences, implies that events are a central construal of timing intervals and temporal experience in general (Zacks and Tversky, 2001). In other words, clocks or similar conventions might be exact in sampling, and apart from questioning what they measure, they are only oscillators that do not count or compare internal and external time (e.g., Buonomano, 2018).

In this somewhat simplified setup, it seems justified to wonder, what if the external environment lacks most or any kind of change perceivable by our modalities, such as in sensory isolation techniques (e.g., floatation-REST/Reduced environmental stimulation therapy) or sensory deprivation techniques (e.g., Ganzfeld technique)? Would human observers still be able to experience the notion of duration under those artificially induced sensory conditions even remote to everyday life? Would the absence of external change disrupt the sense of time? And if it does, how reliably do we derive time estimates from our internal physiological oscillations (e.g., heartbeat, respiratory rate, sleep-wake cycle, neuronal oscillations, etc.)? Not until all these questions are answered will one be able to derive a conclusion about whether the cognitive construction of events or a combination of biological oscillators may provide the ultimate underlying metric of our time perception. For now, we acknowledge that Aristotle's definition of observed time as a relationship between events (“Physics,” specifically in Book IV, chapters 10–14) still stands the trial of time.

## Footnotes

^1^Availability refers to the accessibility of the attentional and memory systems. Available is (i) what is perceivable and (ii) retrievable.

^2^As event segmentation in time perception implies parsing the continuous physical reality into more discrete phenomenology, i.e., separate events, the idea of episodic memory analogously implies the existence of the same segmentation process and generation of the so-called episodes, which are the fundamental memory storage units. As they share the segmentation principle, to avoid potential terminology confusion between “episode” and “event,” an episode is used here to denote a memory of a specific event.
